# Nocardiosis in Immunocompromised Patients on Alternative *Pneumocystis* Prophylaxis

**DOI:** 10.3201/eid2710.210620

**Published:** 2021-10

**Authors:** Alfredo G. Puing, David J. Epstein, Niaz Banaei, Aruna K. Subramanian, Anne Y. Liu

**Affiliations:** City of Hope National Medical Center, Duarte, California, USA (A.G. Puing);; Stanford University School of Medicine, Stanford, California, USA (A.G. Puing, D.J. Epstein, N. Banaei, A.K. Subramanian, A.Y. Liu)

**Keywords:** Nocardia, immunocompromised, trimethoprim/sulfamethoxazole, sulfa, drug allergy, bacteria, fungi, Pneumocystis jirovecii, respiratory infections

## Abstract

Prophylactic trimethoprim/sulfamethoxazole (TMP/SMX) prevents *Pneumocystis jirovecii* pneumonia and nocardiosis in immunocompromised patients but sometimes is avoided because of purported allergies or side effects. Of 25 immunocompromised patients receiving alternative prophylaxis in whom nocardiosis developed, 16 subsequently tolerated TMP/SMX treatment. Clinicians should consider TMP/SMX allergy evaluation and rechallenging to assess patient tolerance.

Trimethoprim/sulfamethoxazole (TMP/SMX) is the drug of choice for *Pneumocystis jirovecii* pneumonia (PJP) prophylaxis in immunocompromised patients (*1*). Second-line prophylactic agents include atovaquone, dapsone, pentamidine, and clindamycin with pyrimethamine. Alternative agents can be less effective than TMP/SMX at preventing PJP and opportunistic infections caused by *Listeria monocytogenes*, *Toxoplasma gondii*, and *Nocardia* spp. Prophylactic TMP/SMX is sometimes avoided because of a prior adverse drug reaction or when patients are receiving drugs that have potentially overlapping toxicities. Nonetheless, second-line PJP prophylaxis regimens can increase the risk for opportunistic infections, such as nocardiosis (*2*). Most nocardiosis occurs in patients with impaired cell-mediated immunity; TMP/SMX is the cornerstone of standard therapy (*3*). We describe a series of nocardiosis cases in immunocompromised patients who were receiving alternative or no PJP prophylaxis because of TMP/SMX avoidance. We provide the reasons for TMP/SMX avoidance and proportion of patients who subsequently tolerated TMP/SMX.

We conducted a retrospective chart review at Stanford Hospital (Stanford, CA, USA) for patients with nocardiosis diagnosed during January 1, 1998–January 28, 2020. We included patients avoiding TMP/SMX for PJP prophylaxis in whom nocardiosis was identified on culture or by molecular techniques, such as 16S rRNA PCR-based assay. We used Stanford Hospital’s protocols for defining immunocompromised status requiring PJP prophylaxis. We collected baseline demographic, clinical, microbiological, and outcome information, including immunocompromising condition, PJP prophylaxis indication and agent, reason for TMP/SMX avoidance, and TMP/SMX rechallenge outcome, if performed. This study was approved by Stanford University’s Institutional Review Board (approval no. 54959).

During the study period, nocardiosis developed among 25 immunocompromised patients deliberately avoiding TMP/SMX. Most (68%) patients were female; median age of patients was 55 years. Among the 25 patients, 7 (28%) were lung transplant recipients, 6 (24%) had undergone allogeneic hematopoietic cell transplantation (HCT), 5 (20%) were heart transplant recipients, and 7 (28%) had other immunocompromising conditions (Appendix Table). At diagnosis, 15 (60%) patients were taking atovaquone, 4 (16%) inhaled pentamidine, 3 (12%) dapsone, and 3 (12%) no antimicrobial drug prophylaxis.

Thirteen (52%) patients were not taking TMP/SMX because of a reported history of allergy, 6 because of concern for cytopenia (24%), and 3 because of elevated creatinine (12%). TMP/SMX was avoided in 1 patient for elevated transaminases, 1 for gastrointestinal intolerance, and 1 for unstated reasons. Among 10 patients with a TMP/SMX allergy label who attempted challenge or desensitization, 7 (70%) tolerated the drug; nonsevere rash developed in the other 3 patients. Among 10 patients avoiding TMP/SMX prophylaxis for nonallergy reasons, 9 (90%) tolerated TMP/SMX when rechallenged. Overall, TMP/SMX introduction was attempted in 20/25 patients; 80% successfully tolerated the drug, and 20% had mild, reversible adverse effects (Figure).

**Figure:**
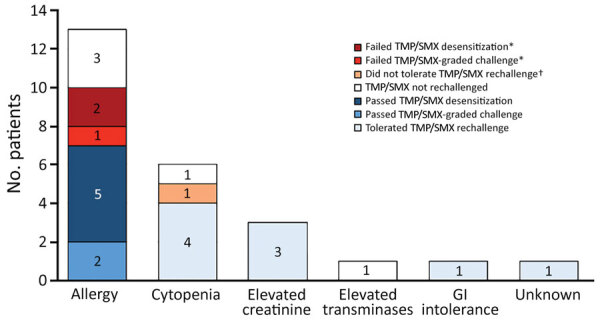
Reasons for TMP/SMX avoidance and TMP/SMX rechallenge outcomes among immunocompromised patients in whom TMP/SMX prophylaxis for *Pneumocystis jirovecii* pneumonia prophylaxis was avoided, Stanford, California, USA. *Failed TMP/SMX introduction because of rash or GI symptoms that were not severe. †Developed intractable nausea and vomiting after TMP/SMX was introduced and did not tolerate rechallenge. GI, gastrointestinal; TMP/SMX, trimethoprim/sulfamethoxazole.

In this retrospective case series, 16/25 (64%) patients who had nocardiosis while deliberately avoiding TMP/SMX prophylaxis ultimately were treated with TMP/SMX. Immunocompromised patients often are prescribed alternative drugs to TMP/SMX prophylaxis because of concerns over side effects or allergic reactions (*4*). However, desensitization or rechallenge could enable a substantial proportion of patients to safely take TMP/SMX for prophylaxis. In our study, 70% of patients with a history of TMP/SMX allergy tolerated a TMP/SMX graded challenge or desensitization when attempted, and 90% of patients avoiding TMP/SMX prophylaxis for nonallergy reasons tolerated TMP/SMX when rechallenged. Our results concur with findings from a study that showed 74% of kidney transplant recipients who underwent TMP/SMX rechallenge had no recurrence of adverse drug reactions (*5*).

TMP/SMX prophylaxis might decrease the incidence of nocardiosis in immunocompromised patients. In a retrospective review of HCT recipients with nocardiosis, most (12/15) cases occurred in patients receiving alternate PJP prophylaxis (*2*). Other studies have questioned the efficacy of TMP/SMX prophylaxis in preventing nocardiosis in HCT or solid organ transplant recipients (*6*–*8*).

Taken together, these findings suggest that rates of this highly pathological infection might be reduced by systematically reevaluating TMP/SMX avoidance and reconsidering prophylactic TMP/SMX. Consulting with an allergist can detect contraindications, such as severe cutaneous adverse reactions, and opportunities for challenge or desensitization. Patients with a history of maculopapular rash, cytopenia, or increased creatinine with TMP/SMX might tolerate reintroduction. Electronic medical records can be designed to prompt revisiting whether TMP/SMX avoidance is appropriate (*9*).

The first limitation of our study is that we only included immunocompromised patients from a single healthcare system; our findings might not be generalizable to other settings. Second, some immunocompromised patients with nocardiosis possibly were not included in our cohort; although defining the incidence of nocardiosis would be informative, the intent of our study was to describe consequences of unnecessary TMP/SMX avoidance. Third, specifics of desensitization or graded challenge protocols were not consistently documented and thus might not be uniform.

Despite these limitations, our study shows that most patients in whom nocardiosis developed while avoiding TMP/SMX prophylaxis later tolerated TMP/SMX treatment. Future research should prospectively evaluate the risks and benefits of TMP/SMX reintroduction in immunocompromised patients who have had a prior adverse reaction. In conclusion, our findings suggest that revisiting TMP/SMX avoidance could prevent nocardiosis cases. 

Appendix Additional information on nocardiosis in immunocompromised patients on alternative *Pneumocystis* prophylaxis.
