# Supplementary material for: Nocardiosis in Immunocompromised Patients on Alternative Pneumocystis Prophylaxis
Source: Emerg Infect Dis. 2021 Oct;27(10):2734–6. doi: 10.3201/eid2710.210620 (PMC8462344; doi:10.3201/eid2710.210620)
Supplement: Appendix — Additional information on nocardiosis in immunocompromised patients on alternative Pneumocystis prophylaxis. [file 21-0620-Techapp-s1.pdf]

Article DOI: https://doi.org/10.3201/eid2710.210620

Nocardiosis in Immunocompromised Patients Receiving Alternative *Pneumocystis* Prophylaxis

Appendix

Appendix Table. Clinical characteristics of nocardiosis and TMP/SMX avoidance among patients treated with alternative prophylactic agents against *Pneumocystis jirovecii* pneumonia*

ID	Age, y/sex	Underlying conditions	Organism	Infection type	Prophylactic agent	Reason TMP/SMX avoided	Allergy testing outcome	Treatment (duration)	Outcome
1	35/F	Dermatomyositis on high-dose systemic steroids	*Nocardia spp*	Pulmonary	Dapsone	Allergy, swollen lips	Did not test or challenge	MIN (6 mo)	S
2	46/M	Anaplastic large cell lymphoma s/p allogeneic HCT	*N. asteroides*	Disseminated (lung and paraspinal)	Inhaled pentamidine	Allergy, rash	Failed desensitization†	CRO (3 mo)	S
3	63/F	COPD s/p lung transplant	*N. farcinica*	Pulmonary	Atovaquone	Cytopenia	Tolerated regular dose reintroduction	TMP/SMX+LZD+MXF (6 mo)	S
4	17/M	Congenital heart disease s/p heart transplant	*N. cyriacigeorgica*	Pulmonary	Atovaquone	Cytopenia	Tolerated regular dose reintroduction	TMP/SMX+CRO+AMK; TMP/SMX+CD (6 mo)	D
5	43/F	LAM s/p lung transplant	*Nocardia spp*	Pulmonary	Atovaquone	Allergy, rash	Failed desensitization†	IPM+LZD; CRO+LZD; LZD; Inh AMK (Unknown)	D
6	51/F	Bronchiolitis obliterans s/p lung transplant	*N. farcinica*	Disseminated (lung and brain)	Inhaled pentamidine	Allergy, rash	Did not test or challenge	MEM+AMC; AMC+CLR; AMC+MIN; DOX+AMC (12 mo)	D
7	55/F	Pulmonary fibrosis s/p lung transplant	*N. cyriacigeorgica*	Pulmonary	Dapsone	Cytopenia	Did not tolerate rechallenge‡	IPM+CIP; MIN (12 mo)	S
8	67/F	COPD s/p lung transplant	*N. cyriacigeorgica*	Pulmonary	Atovaquone	Allergy, itchiness	Passed desensitization	TMP/SMX+MEM (Unknown)	S
9	51/F	Acute myeloid leukemia s/p allogeneic HCT	*N. cyriacigeorgica*	Disseminated (blood, lung, and brain)	Dapsone	Allergy, rash	Passed desensitization	MEM+AMC; MEM; CRO+TMP/SMX; CRO (Unknown)	S
10	79/F	Autoimmune hepatitis on high-dose systemic steroids	*Nocardia spp*	Bacteremia	None	GI intolerance	Tolerated regular dose reintroduction	TMP/SMX (1 wk)	D
11	11/M	Pre-B acute lymphoblastic leukemia s/p allogeneic HCT	*N. cyriacigeorgica*	Mediastinal	Inhaled pentamidine	Allergy, rash	Passed graded challenge	IPM+CRO; MEM+CRO (2 mo)	D

ID	Age, y/sex	Underlying conditions	Organism	Infection type	Prophylactic agent	Reason TMP/SMX avoided	Allergy testing outcome	Treatment (duration)	Outcome
12	17/F	Dilated cardiomyopathy s/p heart transplant	*N. farcinica*	Pulmonary	Inhaled pentamidine	Allergy, rash	Failed graded challenge†	AMC+MIN; MEM+MXF; LZD+MEM; CIP+LZD; CIP+AMC; AMC (10 mo)	S
13	58/M	Dermatomyositis on high-dose systemic steroids	*N. paucivorans*	Pulmonary	Atovaquone	Elevated transaminases	Did not test or challenge	AMC+MIN (Unknown)	S
14	55/F	Adriamycin-induced cardiomyopathy s/p heart transplant	*N. veterana*	Pulmonary	Atovaquone	Cytopenia	Tolerated regular dose reintroduction	TMP/SMX; AMC+MIN; AMC (7 mo)	S
15	63/M	Ischemic cardiomyopathy s/p heart transplant	*N. cyriacigeorgica*	Pulmonary	Atovaquone	Serum creatinine elevation	Tolerated regular dose reintroduction	TMP/SMX; LZD; MEM; ETP+MIN; ETP+MXF;ETP (8 mo)	S
16	59/F	Primary ciliary dyskinesia s/p lung transplant	*N. nova*	Cerebral	Atovaquone	Allergy: desquamating rash	Passed desensitization	IPM+LZD; IPM+LZD+TMP/SMX; MEM+TMP/SMX (3 mo)	S
17	57/M	HIV/AIDS	*N. farcinica*	Pulmonary	None	Allergy: unclear	Passed graded challenge	IPM; TMP/SMX (12 mo)	S
18	56/M	B cell acute lymphoblastic leukemia s/p allogeneic HCT	*N. asiatica*	Pulmonary	Atovaquone	Cytopenia	Tolerated regular dose reintroduction	TMP/SMX (12 mo)	S
19	61/F	Interstitial lung disease s/p lung transplant	*Nocardia spp*	Pulmonary	Atovaquone	Allergy: unclear	Passed desensitization	TMP/SMX; TMP/SMX+MEM; TMP/SMX (6 mo)	S
20	85/M	Non-small-cell lung carcinoma with brain metastases on high-dose systemic steroids	*N. cyriacigeorgica*	Pulmonary	None	None	Tolerated regular dose reintroduction	IPM+TMP/SMX (1 wk)	D
21	30/F	Relapsed Hodgkin lymphoma status post allogeneic HCT	*N. nova*	Pulmonary	Atovaquone	Serum creatinine elevation	Tolerated regular dose reintroduction	TMP/SMX+MEM; TMP/SMX; MEM; TMP/SMX (12 mo)	S
22	44/M	Chronic myelogenous leukemia status post allogeneic HSCT	*N. nova*	Pulmonary	Atovaquone	Serum creatinine elevation	Tolerated regular dose reintroduction	TMP/SMX+IPM; TMP/SMX+CRO (1 wk)	D
23	69/F	Stage IVB follicular lymphoma on high-dose systemic steroids	*N. farcinica*	Disseminated (lung and brain)	Atovaquone	Allergy, drug-induced hypersensitivity syndrome	Did not test or challenge	MEM; IPM+LZD+MXF; MEM+MXF; MEM (10 mo)	D
24	73/F	COPD on high-dose systemic steroids	*N. cyriacigeorgica*	Pulmonary	Atovaquone	Allergy, rash	Passed desensitization	CRO+TMP/SMX; TMP/SMX (Unknown)	S
25	53/F	Transposition of the great arteries, post heart transplant	*N. farcinica*	Pulmonary	Atovaquone	Cytopenia	Did not test or challenge	MXF+LZD+IPM; MXF+IPM; MXF (12 mo)	S

*AMC, amoxicillin/clavulanate; AMK, amikacin; CDN, cefdinir; CIP, ciprofloxacin; CLR, clarithromycin; COPD, chronic obstructive pulmonary disease; CRO, ceftriaxone; D, died; DOX, doxycycline; ETP, ertapenem; GI, gastrointestinal; HCT, hematopoietic stem cell transplantation; inh, inhaled; IPM, imipenem; LAM, lymphangioleiomyomatosis; LZD, linezolid; MEM, meropenem; MIN, minocycline; MXF, moxifloxacin; S, survived.

†Failed TMP/SMX introduction due to rash or GI symptoms that were not severe.

‡Developed intractable nausea and vomiting after TMP/SMX was introduced.
